# Early loss of ambulation is not a representative clinical feature in Duchenne muscular dystrophy dogs: remarks on the article of Barthélémy et al.

**DOI:** 10.1242/dmm.019216

**Published:** 2015-03

**Authors:** Dongsheng Duan, Chady H. Hakim, Carlos E. Ambrosio, Bruce F. Smith, H. Lee Sweeney

**Affiliations:** 1 Department of Molecular Microbiology and Immunology, School of Medicine, The University of Missouri, Columbia, MO 65212, USA; 2 Department of Neurology, School of Medicine, The University of Missouri, Columbia, MO 65212, USA; 3 Department of Veterinary Medicine, Faculty of Animal Science and Food Engineering, University of São Paulo, Pirassununga 05508-070, Brazil; 4 Scott-Ritchey Research Center, College of Veterinary Medicine, Auburn University, Auburn, AL 36849, USA; 5 Department of Pathobiology, College of Veterinary Medicine, Auburn University, Auburn, AL 36849, USA; 6 Department of Physiology, School of Medicine, The University of Pennsylvania, Philadelphia, PA 19104, USA

## Remarks on the article of Barthélémy et al.: Predictive markers of clinical outcome in the GRMD dog model of Duchenne muscular dystrophy

Dystrophin-deficient dogs are the most commonly used large animal model for Duchenne muscular dystrophy (DMD), a lethal muscle disease currently without an effective therapy. Tremendous progress has been made over the last few years in the development of novel pharmacological and genetic therapies for DMD. Validation of these exciting findings in DMD dogs will pave the way to future clinical tests in affected humans. Unfortunately, our understanding on disease progression in affected dogs remains limited. To better characterize the natural history of the disease in dogs, Barthélémy et al. studied golden retriever muscular dystrophy (GRMD) dogs in their colony (Barthélémy et al., 2014). In the GRMD dog, dystrophin expression is abolished owing to a point mutation in intron 6 of the dystrophin gene (Cooper et al., 1988). Sixty-one GRMD dogs were followed starting from 2 months of age. By the age of 6 months, 15 dogs (24.59%) lost ambulation. These dogs were classified as the severe form. Two additional dogs lost ambulation at ~7.3 months. The remaining 44 dogs were ambulant throughout their lives and were classified as the mild form. A comparison of the blood and gait data at the beginning of the study (when dogs were 2 months old) identified three biomarkers that, when used together, can accurately predict the phenotype (mild or severe) that the dogs will have at 6 months of age. Specifically, an increase of peripheral CD4^+^CD49d^Hi^ T cells, a decrease of the spontaneous gait speed and a reduction of the stride frequency were found to associate with early loss of ambulation. The results of this study have important implications in designing preclinical studies in dogs. For example, if a treatment can prevent the early loss of ambulation in dogs with severe-type disease, it might suggest that the candidate treatment has the therapeutic value.

When GRMD dogs were initially characterized in the late 1980s, Valentine et al. pointed out that a “complete loss of ambulatory function, which occurs in all DMD patients, is not a feature of CXMD (canine X-linked muscular dystrophy)” (Valentine et al., 1988). To determine whether the loss of ambulation at a young age is a clinical marker for dystrophin-deficient dogs in general, we reviewed data from four different DMD colonies that are located in Brazil and the USA (Table 1). These dogs carry different mutations in the dystrophin gene and are on different strain backgrounds (including GRMD) (Table 1). Although a high neonatal mortality rate (17–37%) was noted, as initially reported by Valentine et al. (28%), we did not see a high rate of ambulation loss at 6 months of age (Table 1). From a total of 380 affected dogs, only one dog (0.26%) lost its walking ability by the age of 6 months. Our data suggest that there are important phenotypic differences in different DMD dog colonies. Currently, dystrophin deficiency has been reported in more than 20 dog breeds (McGreevy et al., 2015). In addition to the colonies mentioned in this paper (Table 1), experimental DMD dog colonies have also been established in a number of other institutions in Australia, Japan, the United Kingdom and the USA (McGreevy et al., 2015). The age of ambulation loss in affected dogs in these colonies has not been reported. It is possibly that variations between the colony located at the Veterinary School of Alfort, France (Barthélémy et al., 2014) and the four colonies we have surveyed (Table 1) could exist. Future studies are needed to gain the consensus and to identify the factors that might have contributed to the inter-colony variation (such as the genetic background of the strain, the level of inbreeding and the specific type of dystrophin gene mutation). In the meantime, caution should be taken when interpreting and extrapolating the ambulation data observed in the French colony. Additional multicenter studies are warranted to establish a solid baseline to guide translational study using the canine model.

**Table 1.**
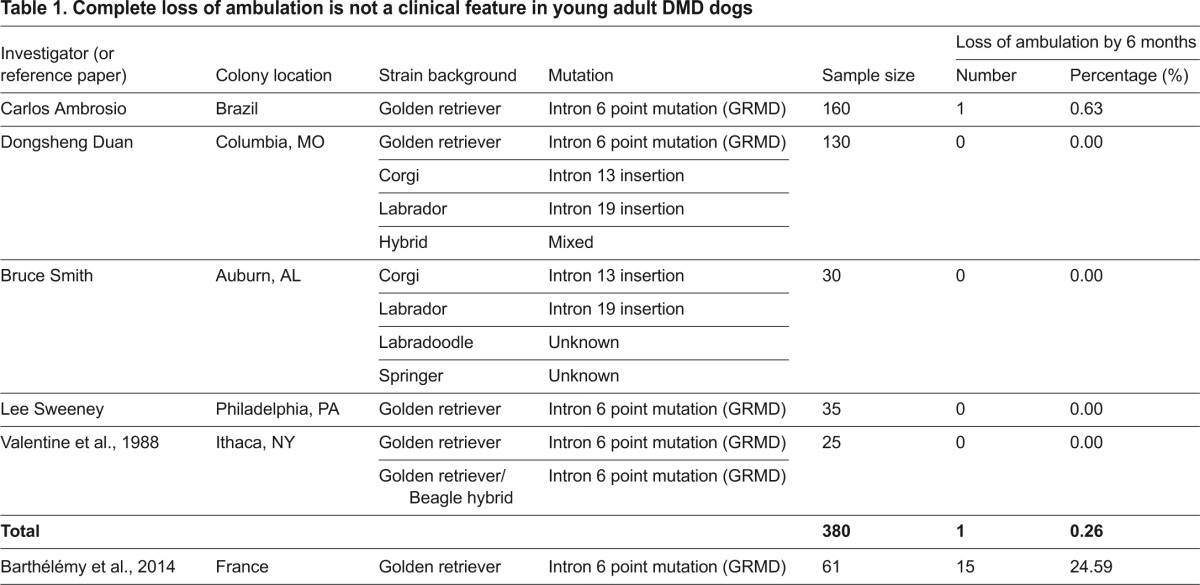
Complete loss of ambulation is not a clinical feature in young adult DMD dogs
